# Supplementary material for: Structural determinants of lipid specificity within Ups/PRELI lipid transfer proteins
Source: Nat Commun. 2019 Mar 8;10:1130. doi: 10.1038/s41467-019-09089-x (PMC6408443; doi:10.1038/s41467-019-09089-x)
Supplement: Supplementary file 1 — Supplementary Information [file 41467_2019_9089_MOESM1_ESM.pdf]

Structural determinants of lipid specificity within Ups/PRELI lipid transfer proteins

X. Miliara, T. Tatsuta, J-L. Berry et al.

SUPPLEMENTARY INFORMATION

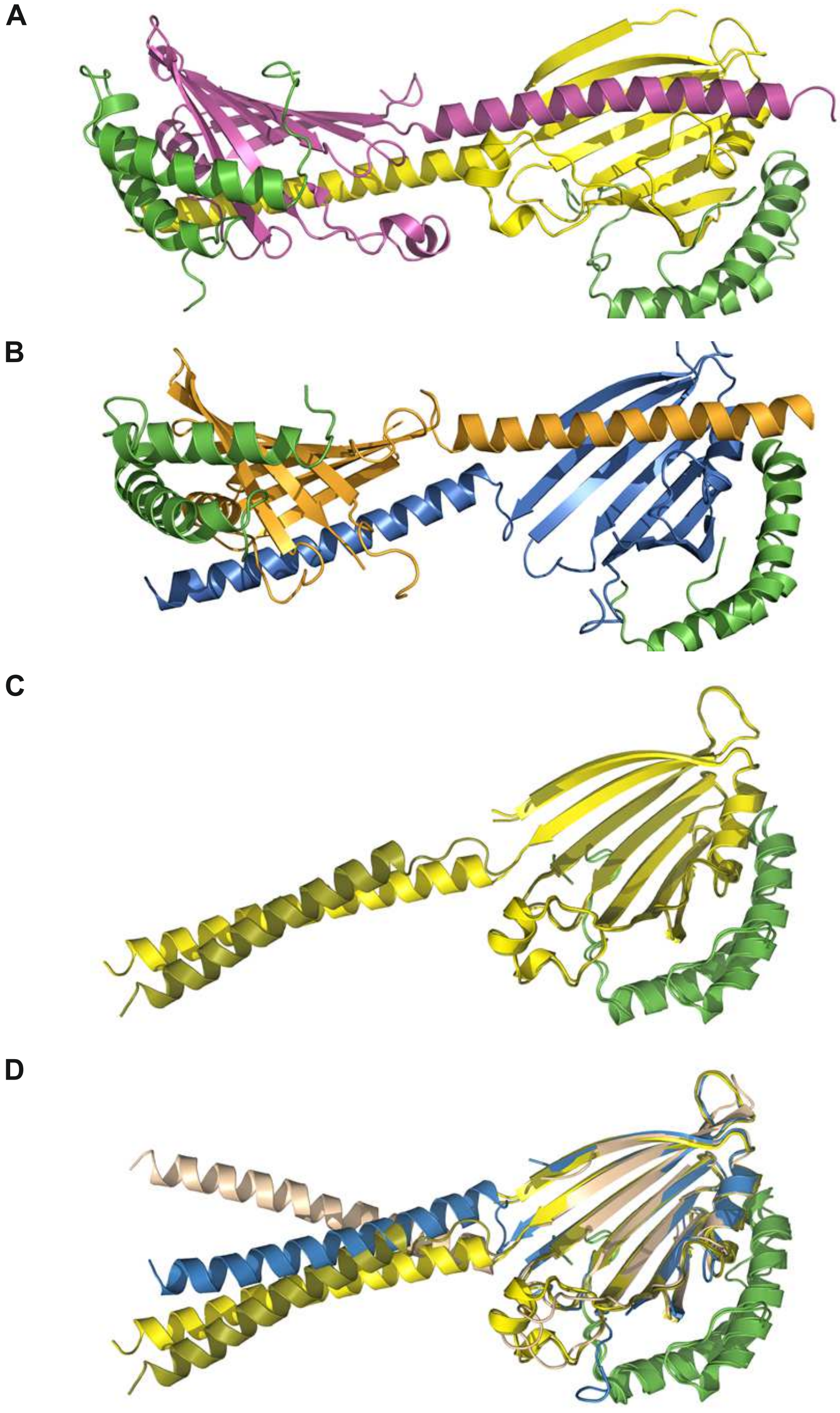

Supplementary Figure 1. Structural comparison of the domain swapped heterotetermers obtained from crystal structures of the PRELI family (corresponds to Fig. 1)

A) PRELID1$^{1\text{-}173}$-TRIAP1, B) PRELID3b-TRIAP1, C) Superposition of PRELID1$^{1\text{-}173}$-TRIAP1 and PRELID3b-TRIAP1 protomers on the core PRELI domain and D) Superposition of one PRELID1-TRIAP1, PRELID3b-TRIAP1 and Ups1-MDM35 protomers on the core PRELI domain.

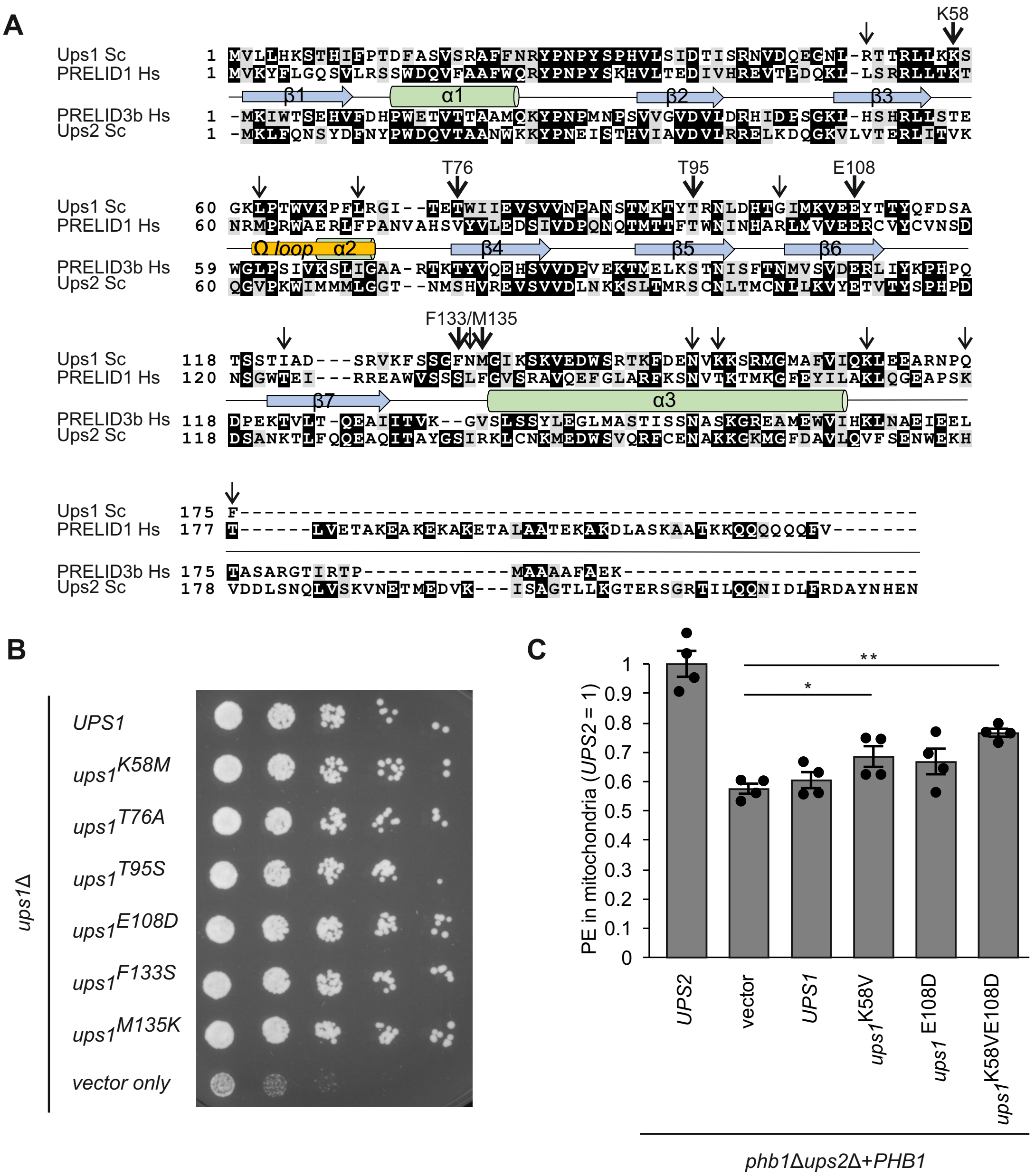

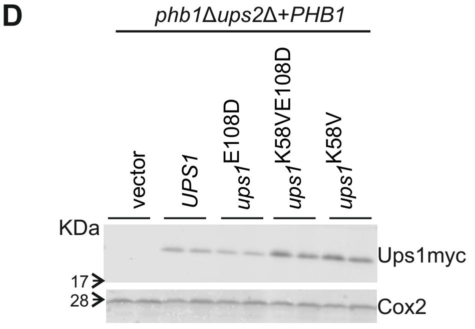

Supplementary Figure 2. Characterisation of Ups1 variants substituting for Ups2 (corresponds to Fig. 2)

A) Multiple sequence alignment of yeast (Sc) Ups1, yeast Ups2, human (Hs) PRELID1 and human PRELID3b (SLMO2) with secondary structure elements. Arrows highlight amino acids that were found to be most frequently mutated in Ups1. Bold arrows indicate amino acids that were mutated in >10 Ups1 variants, whereas thin arrows point to residues found to be mutated in 5 to 10 Ups1 variants. B) Growth of *ups1*Δ cells expressing *UPS1* or the indicated mutants thereof. Cells were grown on selective medium lacking leucine and inositol in the presence of glucose. C) PE levels in mitochondria isolated from *ups2*Δ*phb1*Δ cells harbouring plasmid-borne *PHB1* and a plasmid encoding the indicated *UPS1* variants or *UPS2*. Values are normalized to the level of PE in mitochondria from cells expressing *UPS2*. Error bars represent mean ± SEM, n = 4. * $p<0.05$, ** $p<0.01$. D. Levels of Ups1 and Ups1 variants in mitochondrial fraction used for the assessment of PE levels in C. 50 µg protein were analyzed by SDS-PAGE and immunodetection by anti-myc. Cox2 levels are shown as a control.

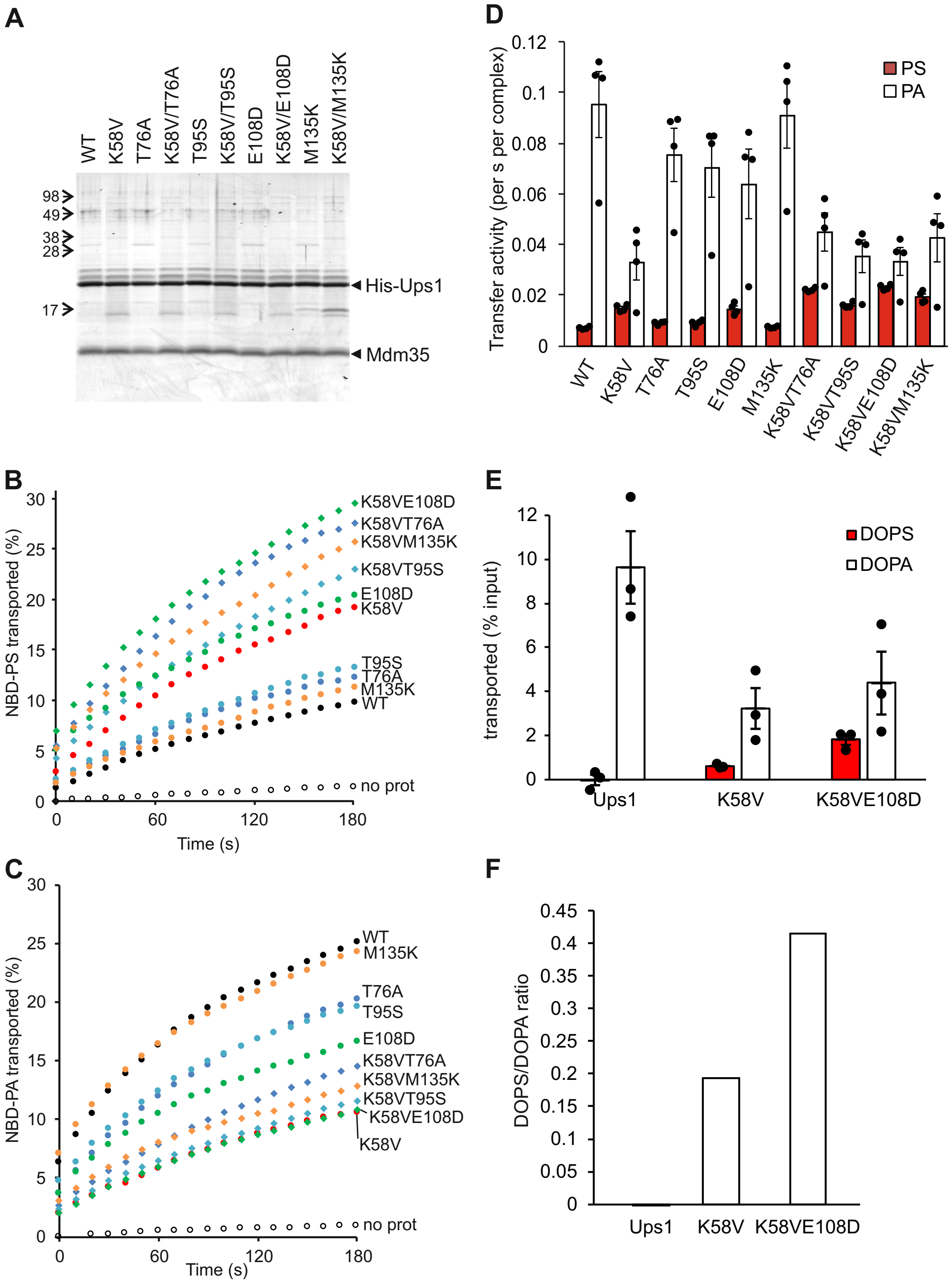

Supplementary Figure 3. Lipid transfer activities of Ups1 mutants *in vitro* (corresponds to Fig. 3)

A) Tris-Tricine-PAGE analysis of recombinant Ups1-Mdm35 complexes (containing hexyahistidine-tagged Ups1) and mutant variants thereof (20 pmol) after expression in *E. coli* and purification by metal chelating chromatography. The gels were stained with Coomassie blue. B) PS transfer and C) PA transfer by purified Ups1-Mdm35 complexes and variants thereof. D) Specific PS (red bars) and PA (white bars) transfer activities of Ups1-Mdm35 complexes and mutants. Error bars represent mean ± SEM, n = 4. E. Lipid transfer specificity of Ups1 and its mutant variants assessed by non-fluorescent, endogenous PA (DOPA) and PS (DOPS). Donor liposomes (12.5 µM; DOPC/DOPE/Lac-PE/DOPA/DOPS/17:0 PE/NBD-PE = 48/34.6/5/5/5/2/0.4%) and acceptor liposomes (50 µM; DOPC/DOPE/Lac-PE/POPA/POPS/17:0 PC/Rhodamine-PE = 48/34.9/5/5/5/2/0.1%) were incubated with lipid transfer proteins (25 nM) for 3 min. After isolation of acceptor liposomes by flotation centrifugation, lipids transferred were quantified by qMS and shown as a percentage of input. See methods for experimental details. N=3. Error bars represent mean ± SEM. F) DOPS/DOPA transfer ratio of E).

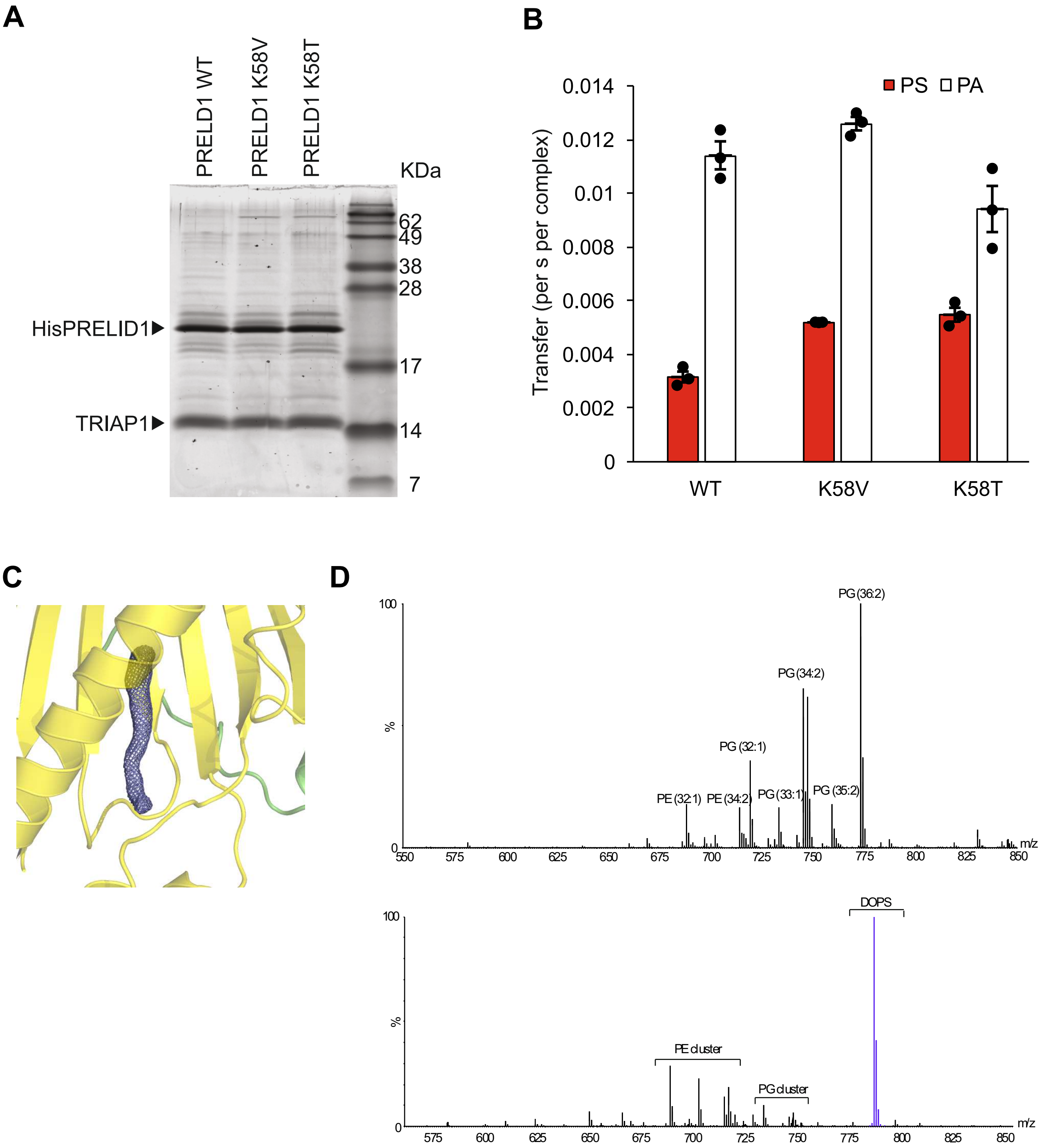

Supplementary Figure 4. Structural and functional characterisation of human PRELI-like lipid transfer proteins (corresponds to Fig. 4)

A) Tris-Tricine-PAGE analysis of recombinant PRELID1-TRIAP1 complexes (containing hexahistidine-tagged PRELID1) and variants thereof (40 pmol), which were used in lipid transfer assays *in vitro*. B) PS (red bars) and PA (white bars) transfer activities of PRELID1-TRIAP1 complexes and mutants. Error bars represent mean ± SEM, n = 3. C) Example electron density map of *E. coli* derived lipid bound to PRELID1 purified from *E. coli*. The F_o–F_c difference Fourier map was calculated by omitting the bound molecule, and is shown with blue meshes countered at 1.0σ. D) Identification of lipid content in PRELID1-K58V mutant using Mass spectrometry. Analysis immediately before (top) and after (bottom) phospholipid transfer reaction with PS-enriched liposome with the PRELID1-K58V mutant.

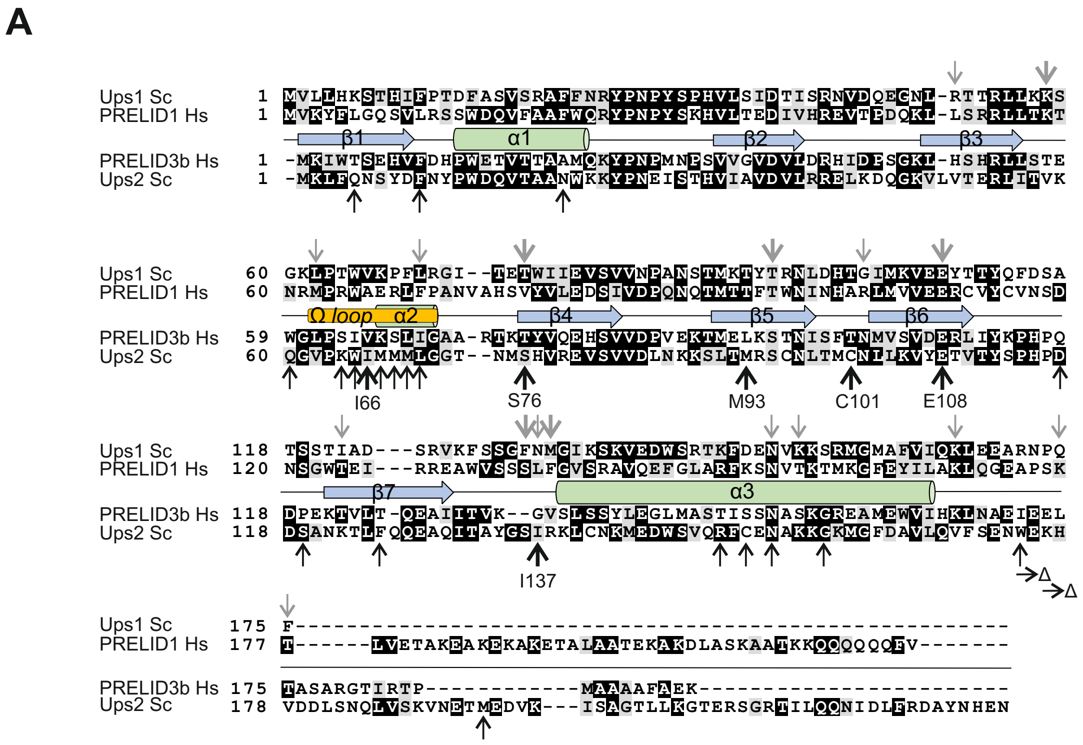

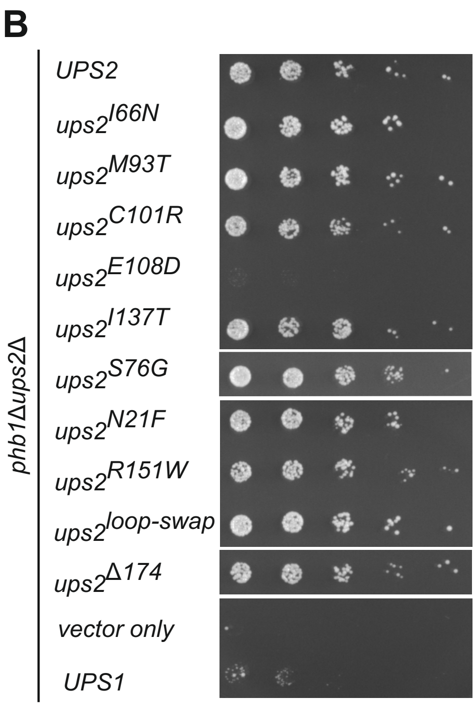

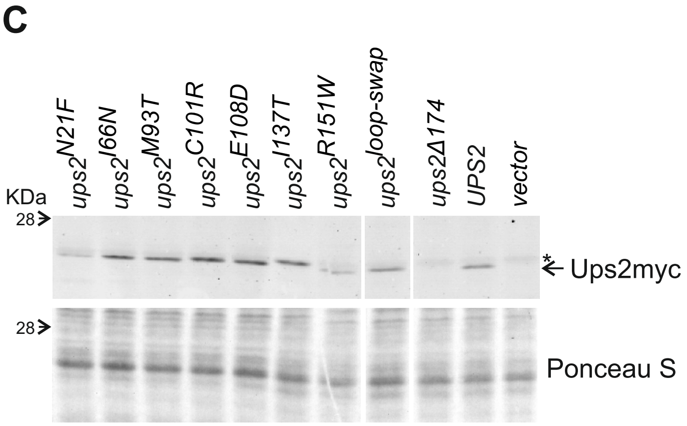

Supplementary Figure 5. Characterisation of Ups2 variants substituting for Ups1 (corresponds to Fig. 5)

A) Multiple sequence alignment among yeast (Sc) Ups1, yeast Ups2, human (Hs) PRELID1 and PRELID3b (SLMO2) with secondary structure elements. Arrows highlight amino acids that were found to be most frequently mutated in Ups2. Bold arrows indicate amino acids found in >10 Ups2 variants, whereas thin arrows point to amino acids found in 5 to 10 variants. B) Growth of *phb1Δups2Δ* cells expressing *UPS1, UPS2* or the indicated mutants of *UPS2* on selective medium lacking leucine in the presence of glucose. C) Levels of Ups2 and Ups2 variants in transformants used in Fig. 5C. 50 µg protein of total membrane fraction were analyzed by SDS-PAGE and immunodetection by anti-myc. Ponceau S staining of the blot membrane was shown to control loading. *non-specific signal. Ups2Δ174 does not have Myc tag because of truncation.

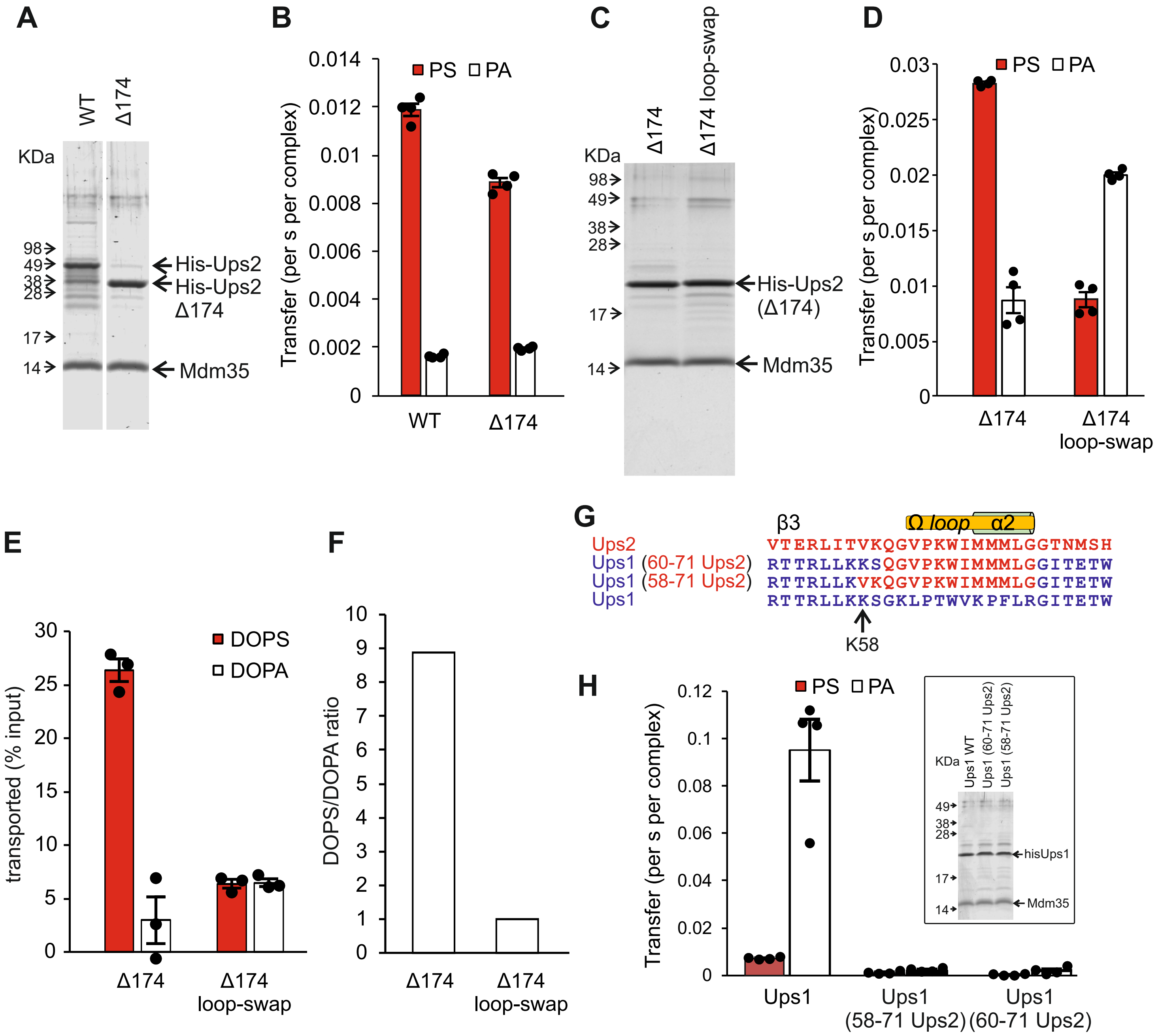

Supplementary Figure 6. Characterisation of the loop-swap variant of Ups2 *in vitro* (corresponds to Fig. 6)

A) Tris-Tricine-PAGE analysis of recombinant Ups2-Mdm35 complexes containing hexahistidine-tagged Ups2 and, when indicated, a truncated Ups2 variant lacking the C-terminal 58 amino acids (Δ174, 20 pmol). B) PS (red bars) and PA (white bars) transfer activities of Ups1-Mdm35 complexes and complexes harbouring C-terminal truncated Ups2. Error bars represent mean ± SEM, n = 4. C) Tris-Tricine-PAGE analysis of recombinant Ups2Δ174-Mdm35 complexes containing Ups2Δ174 or the loop-swap variant (carrying amino acids 60-71 of Ups1) thereof (used in Fig. 6). D) PS- (red bars) and PA- (white bars) transfer activities of Ups1Δ174-Mdm35 complexes and the loopswap variant thereof. Error bars represent mean ± SEM, n = 4. E) Lipid transfer specificity of Ups2 and its loop-swap variant assessed by non-fluorescent, endogenous PA (DOPA) and PS (DOPS). Donor liposomes (12.5 µM; DOPC/DOPE/CL/Lac-PE/DOPA/DOPS/17:0 PE/NBD-PE = 48/19.6/15/5/5/5/2/0.4% reconstituted in assay buffer containing 12.5% sucrose) and acceptor liposomes (50 µM; DOPC/DOPE/CL/Lac-PE/POPA/POPS/17:0 PC/Rhodamine-PE = 48/19.9/5/5/5/2/0.1%, reconstituted in assay buffer without sucrose) were incubated with lipid transfer proteins (80 nM) for 5 min. After isolation of acceptor liposomes by flotation centrifugation, lipids transferred were quantified by qMS and shown as a percentage of input. N=3. Error bars represent mean ± SEM. F) DOPS/DOPA transfer ratio of E). G) Amino acid sequence of the region surrounding the Ω loop in Ups1 and Ups2 and of the Ups1 loop-swap mutants. H) PA and PS transfer activities of Ups1 and loop-swap variants. Recombinant protein complexes (12.5 nM) were incubated with donor liposomes (12.5 µM) containing NBD-PS or PA and Rhodamine-PE (PC/PE/CL/Lac-PE/NBD-PS or NBD-PA/Rhodamine-PE = 50/23/15/5/5/2 mol%) and acceptor liposomes (50 µM; PC/PE/CL/Lac-PE/PA or PS = 50/25/15/5/5 mol%) at 25 °C. Lipid transfer activities were calculated from the initial slope of the increase of NBD fluorescence. Error bars represent mean ± SEM, n = 4. Inlet: assessment of the quality of purified Ups1-Mdm35 complexes used in the assay by SDS-PAGE and Coomassie staining.

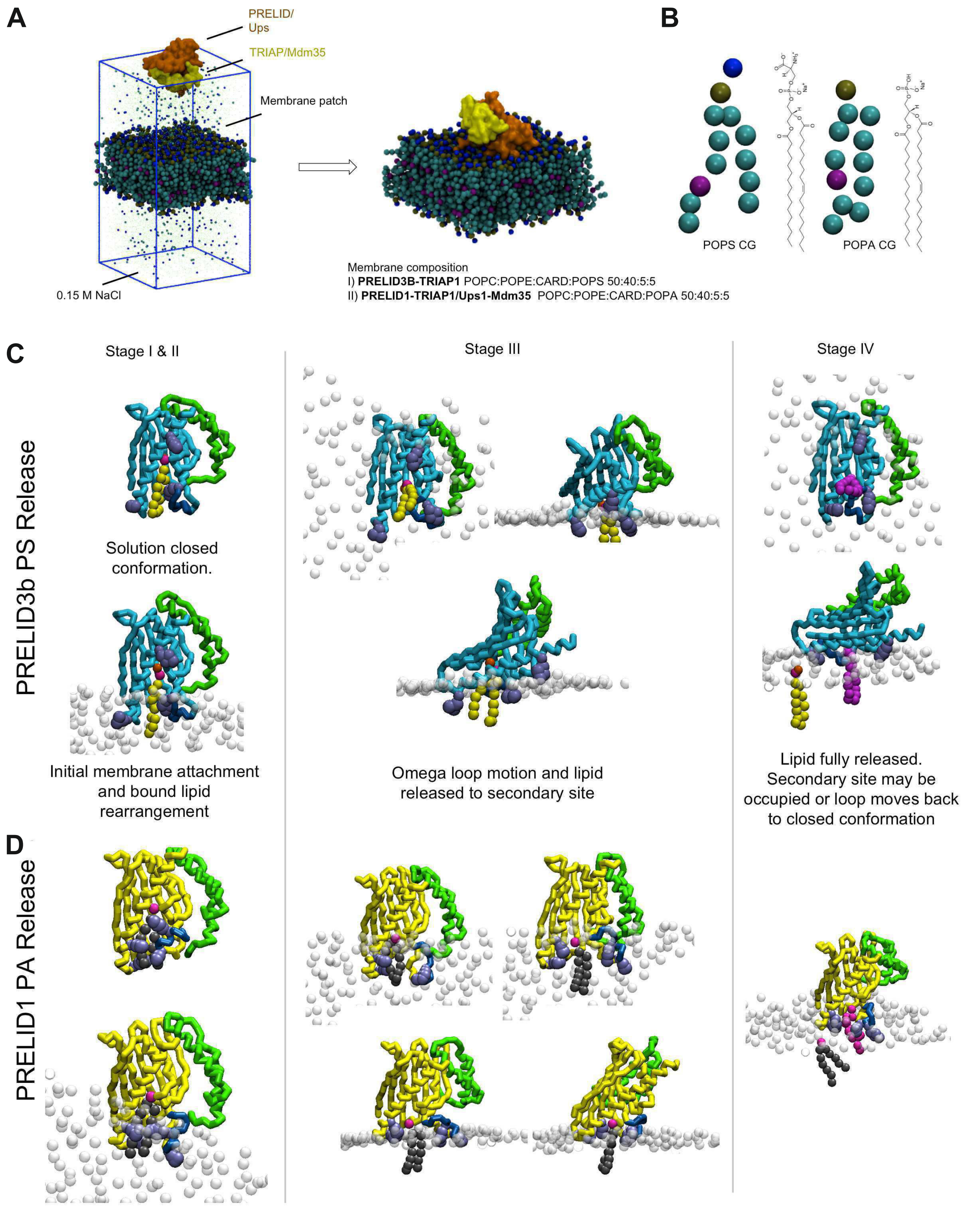

Supplementary Figure 7. Stages of membrane binding and lipid release of PRELID-TRIAP complexes.

A) Simulation set up including membrane composition. B) Approximate mapping of coarse grain lipid particles of POPS and POPA. C) Stages of membrane binding and lipid release of PRELID3b-TRIAP1, coloured according to Supplementary Movie 1. D) Stages of membrane binding and lipid release of PRELID1-TRIAP1, coloured according to Supplementary Movie 2.

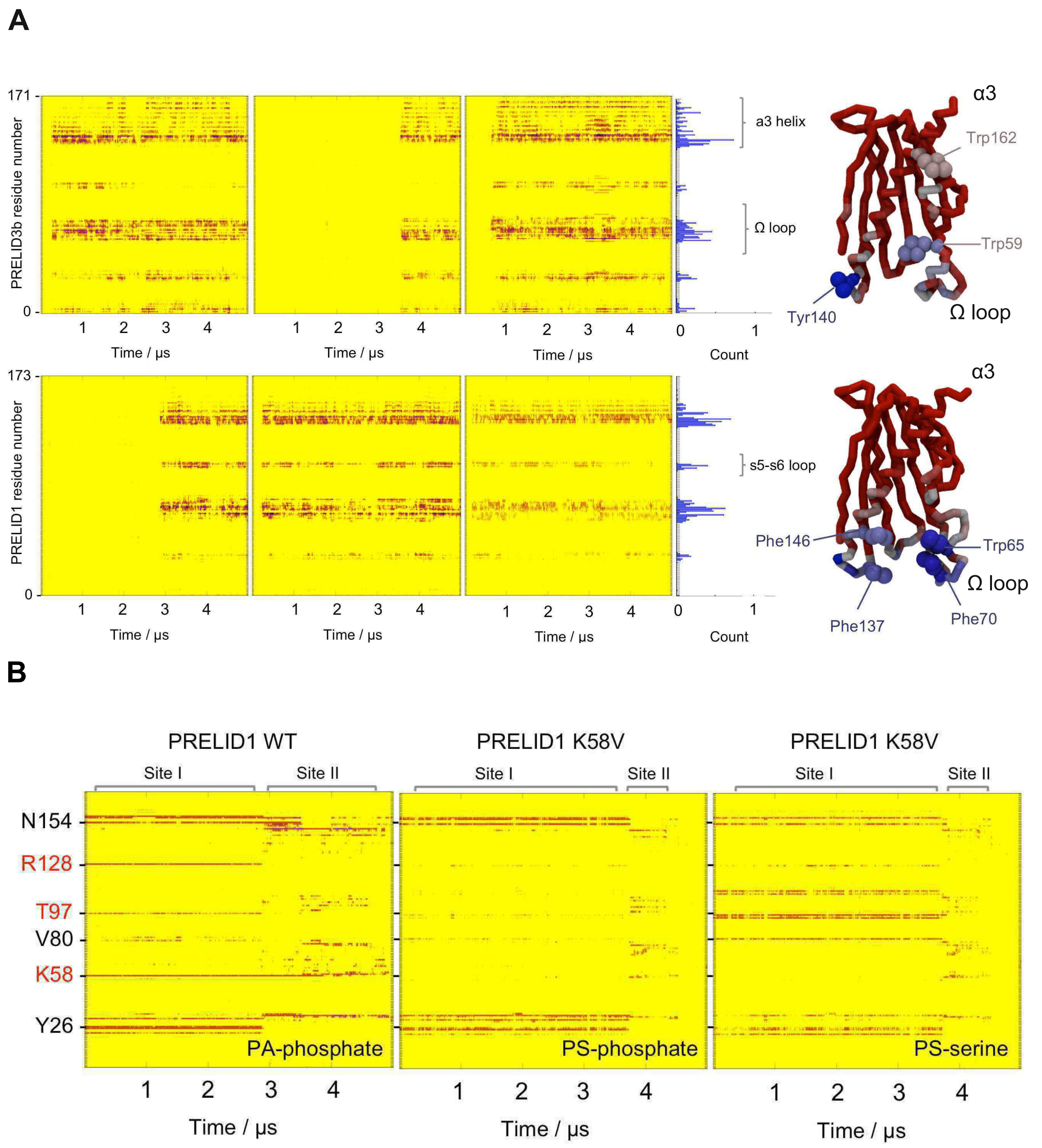

Supplementary Figure 8. Stability of membrane bound state of PRELID3-TRIAP1 and PRELID1-TRIAP1 and key interactions with lipid substrate of PRELID1 wt and K58V mutant.

A) Heatmaps indicate the key contacts of PRELID3b (upper) and PRELID1 (lower) with the hydrophobic region of the membrane (lipid tail particles). Once the complex binds to the membrane it remains bound for the duration of the simulation, with the same orientation for all 3 repeats. The degree of burial into the hydrophobic portion of the membrane, averaged over the 3 simulations, is shown by mapping on the protein backbone trace (red, no contacts, blue, maximum). For PRELID3b, residues in the a3 helix are buried whilst for PRELID1 the more upright orientation brings the s5-s6 loop into contact with the lipid tails, as well as greater proportion of simulation time spent buried for the aromatic hydrophobic residues indicated. B) Interactions of PA phosphate group with the PRELID1 wt (left) and PS phosphate (middle) and serine (right) particles with PRELID1 K58V mutant. Residues that show key interaction in the wt PA but are lost in K58V PS are highlighted in red.

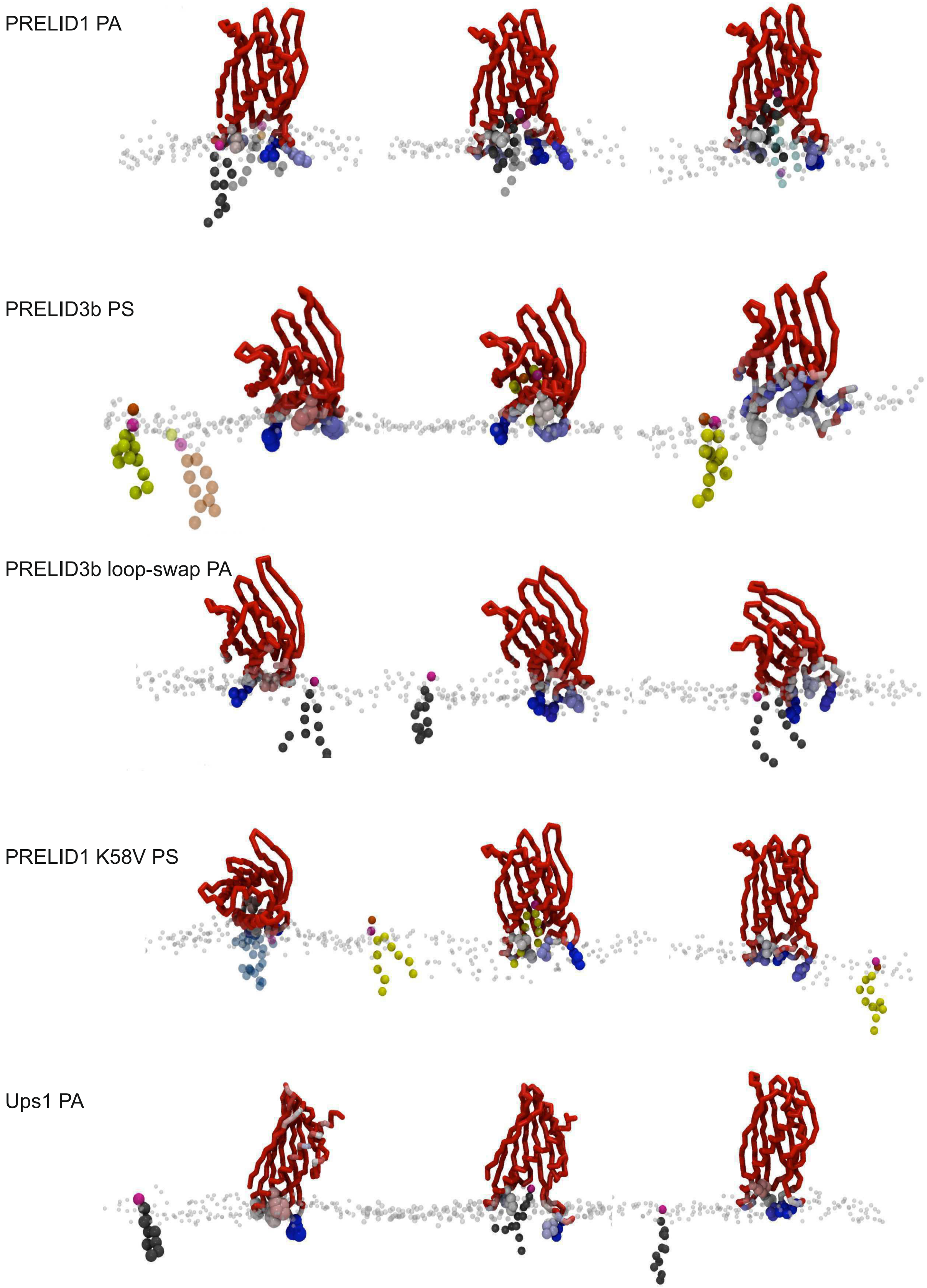

Supplementary Figure 9. Orientation and membrane anchoring of PRELID-TRIAP complexes

Snapshots corresponding to the final frames of each set of simulations (Supplementary Table 6). In all cases the PRELID/Ups1 backbone is shown as a trace coloured according to burial within the membrane - number of contacts with lipid tail particles averaged per trajectory. Bulky hydrophobic Tyr, Trp, Phe residues buried in the membrane are shown as spheres. These snapshots highlight the range of motions of the omega loop, the relative orientations of PRELID3b vs PRELID1/Ups1, and the stochastic nature of the membrane binding and lipid release process. In cases where the original bound lipid has been released and a new lipid occupies the secondary binding site, the new lipid is shown as transparent spheres

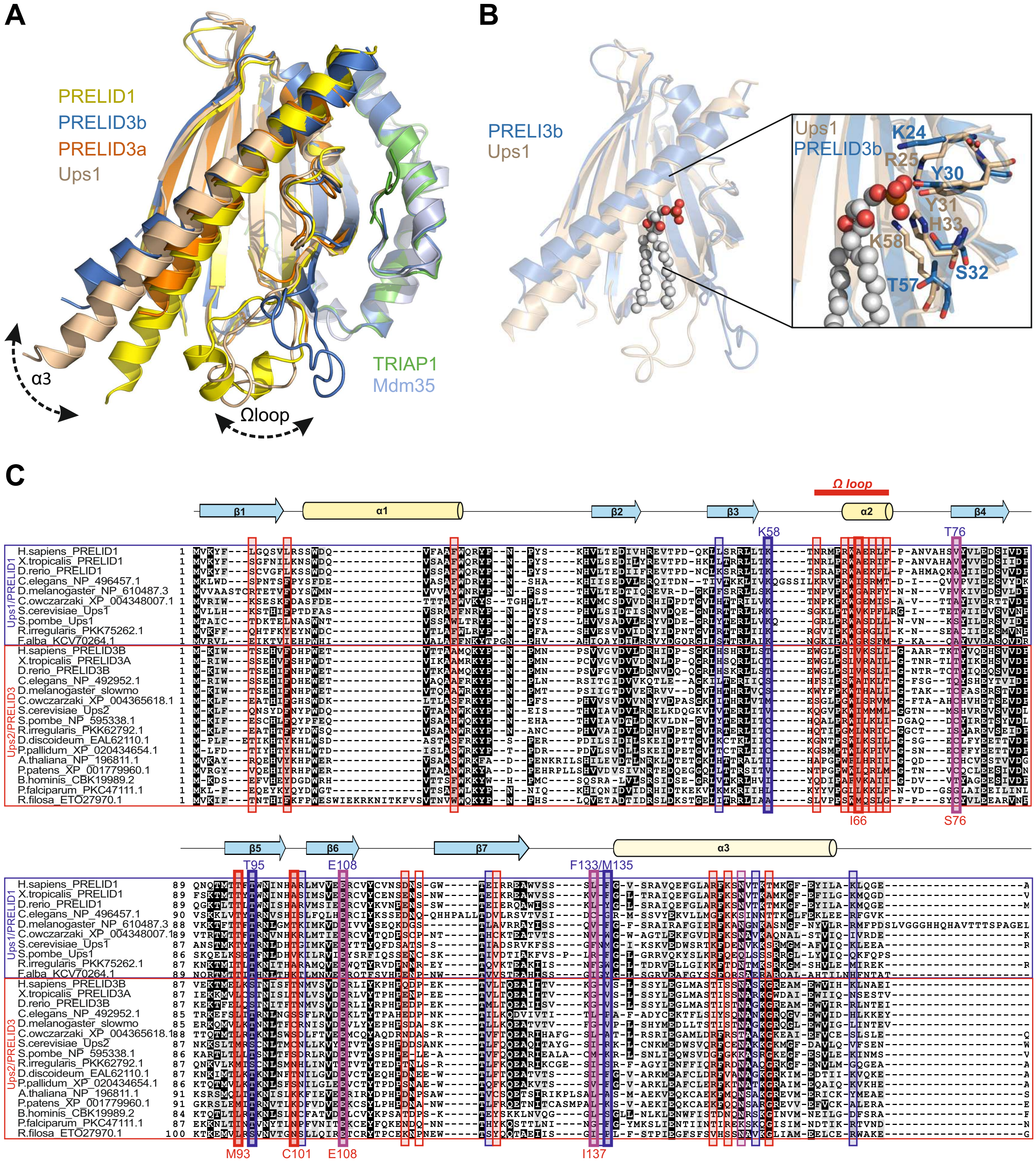

Supplementary Figure 10. Structural comparison of the PRELI family

A) Structural comparison of yeast Ups1 and human PRELID1, PRELID3b and PRELID3a. Superposition of PA-bound Ups1-Mdm35 structures (pdb:4ytx; model coloured in beige for Ups1, grey for PA monomer with light blue for Mdm35)[1], PRELID1, PRELID3b, PRELID3a (pdb:4xzv)[2]. The structural alignment shows the flexible nature of the Ω loop and the C-terminal α-helix of the PRELI domain. Dotted arrows indicate the change of the position of these flexible regions between the four homologs. B) Comparison of amino acid residues involved in head group interactions between PA-specific Ups1 and PS-specific PRELID3b. Left; Superposition of PRELID3b-TRIAP1 (blue-green) and PA-bound Ups1-Mdm35 (beige-grey-light blue). Right; zoom in view of residues identified in interactions with negatively charged head group of PA are shown in sticks. Residues at equivalent positions are shown in blue sticks on PRELID3b which is specific for PS. All residues are numbered according to the protein sequence. C) Multiple sequence alignment of eukaryotic Ups/PRELI lipid transfer proteins using T-Coffee. Amino acids allowing PS transfer by Ups1 when mutated are marked by blue boxes, whereas residues promoting PA transfer by Ups2 when mutated are marked by red boxes. Variants of Ups1 or Ups2 in amino acid E108, T76/S76 and F133/I136 (of Ups1/Ups2) were found in both genetic screens and are marked by purple boxes.

Supplementary Figure 2C

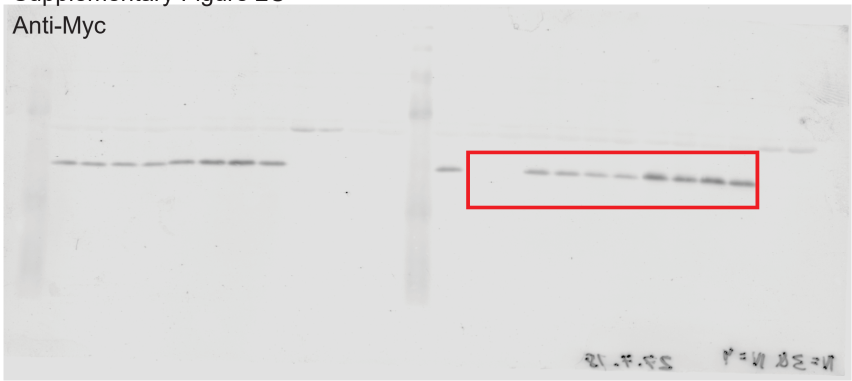

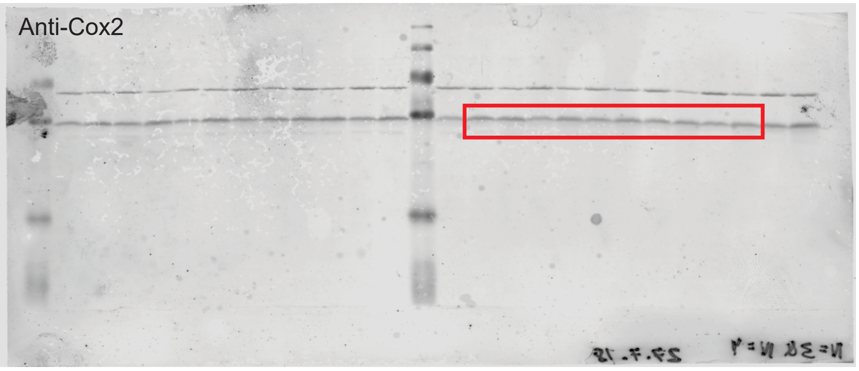

Supplementary Figure 3A

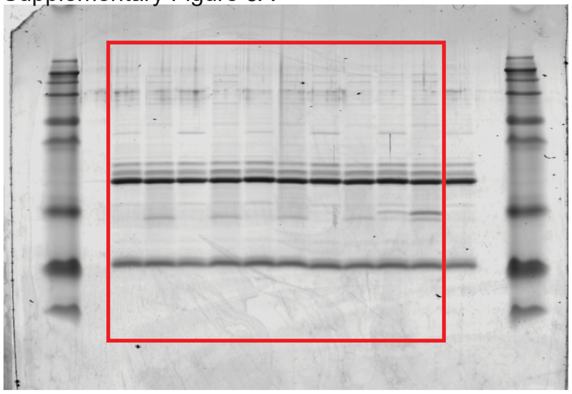

Supplementary Figure 4A

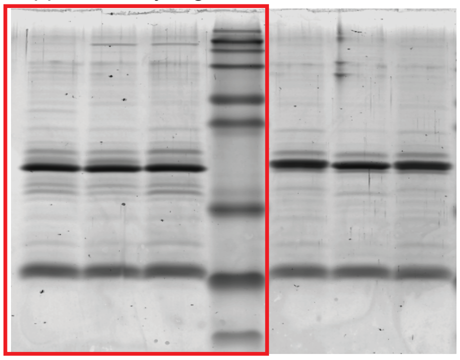

Supplementary Figure 5C

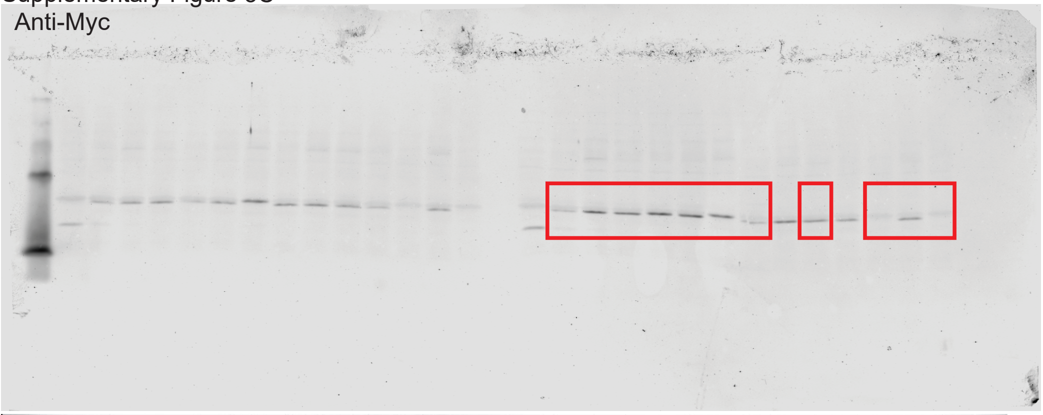

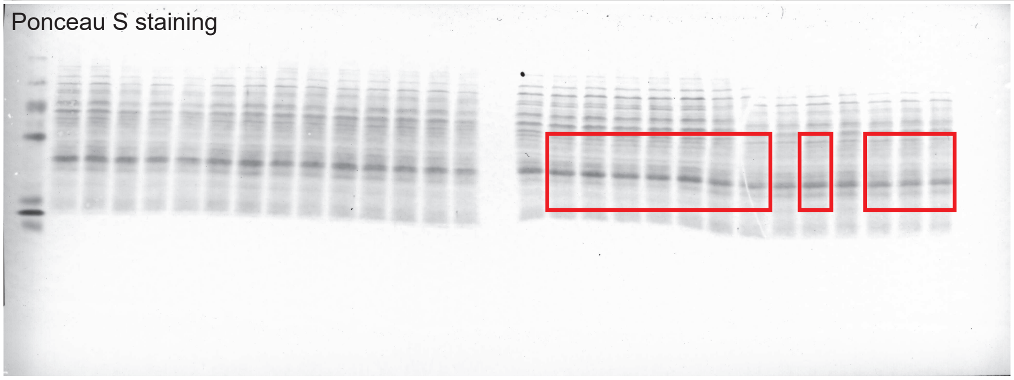

Supplementary Figure 6C

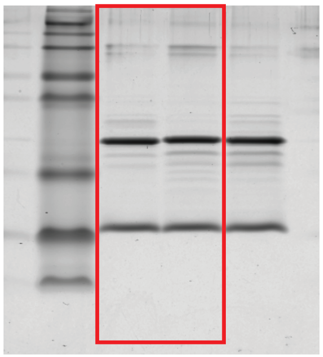

Supplementary Figure 6H

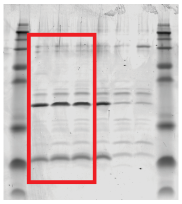

Supplementary Figure 6A

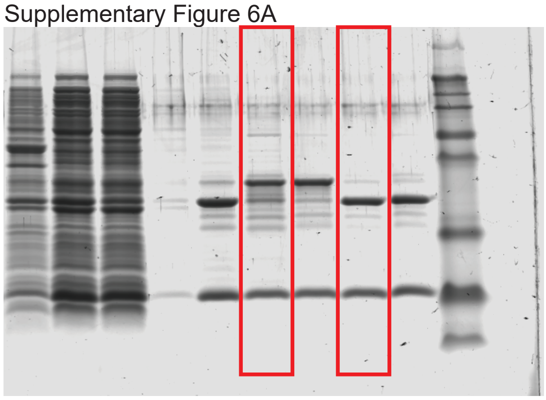

Supplementary Figure 11. Uncropped gel images used in the study
Red rectangles represent cropping positions to create panels in indicated figures.

Supplementary Table 1. Base exchanges found in the reverse genetic screen of Ups1

Residue	Total counts	Exchanged to	in Ups2
**K58**	24	M (21), R (2), E (1)	V
**T76**	17	A (17)	S
**T95**	11	S (7), A (3), I (I)	S
**E108**	11	D (8), V (2), A (1)	E
**F133**	12	L (7), S (3), Y (2)	(S)
**M135**	11	K (5), T (3), V (2), I (1)	(R)

Supplementary Table 2. Base exchanges found in the reverse genetic screen of Ups2

Residue	Total counts	Exchanged to	in Ups1
**I66**	12	N (6), T (3), M (1), S (1), V (1)	V
**S76**	18	C (8), G (6), I (4)	T
**M93**	11	T (13), V (5)	T
**C101**	13	R(12), S(1)	T
**E108**	13	G (5), V (4), D (2), A (1), Q (1)	E
**I137**	26	T (17), N (7), V (2), S (2)	(N)

Supplementary Table 3. Hydrophobicity in α2 calculated by Kyte & Doolittle scale

	Ups1/PRELID1	Ups2/PRELID3b
S. cerevisiae	0.529	1.314
H. sapiens	-1.343	1.643
D. rerio	-0.629	1.557
C. elegans	-0.714	-0.200
D. melanogaster	-0.914	0.200
C. owczarzaki	-0.443	0.471
R. irregularis	-0.257	0.614

Supplementary Table 4. Summary of simulations performed.

#	Protein	Mutant	Re-peat	Bound Lipid	Membrane bind (µs)	Lipid release time (µs) or final position	Occupancy Site II	Final status of Ω-loop
1	PRELID3b	wt	1	POPS	0.26	2.13	2.5 – 2.9 µs POPE	Closed
2	PRELID3b	wt	2	POPS	3.53	Site I	-	Closed
3	PRELID3b	wt	3	POPS	0.64	1.32	1.3 – 1.8 µs POPE 2.9 – 3.6 µs POPC	Open
4	PRELID1	wt	1	POPA	2.87	4.69	4.9 – 5.0 µs POPE	Open
5	PRELID1	wt	2	POPA	4.07	Site II	4.5 - 5.0 µs POPA	Open
6	PRELID1	wt	3	POPA	0.17	Site I	0.9 – 3.9 µs CARD 4.0 – 5.0 µs POPA	Open
7	PRELID3b	Loop-swap	1	POPA	0.24	2.42	-	Closed
8	PRELID3b	Loop-swap	2	POPA	0.26	0.61	-	Closed
9	PRELID3b	Loop-swap	3	POPA	0.25	2.56	-	Closed
10	PRELID1	K58V	1	POPS	0.84	1.62	-	Closed
11	PRELID1	K58V	2	POPS	0.28	Site I	-	Closed
12	PRELID1	K58V	3	POPS	1.40	4.40	4.4 - 4.7 µs POPE 4.7 - 5.0 µs POPE	Open
13	Ups1	wt	1	POPA	1.41	2.25	-	Closed
14	Ups1	wt	2	POPA	4.04	Site II	-	Closed
15	Ups1	wt	3	POPA	1.34	4.34	-	Closed

The time taken to bind the membrane in each case as well as the time to fully release the bound lipid, or the final location of the lipid if not released. Site 1 and Site 2 correspond to those shown in Figure 7. The omega loop was referred to as closed if the smallest distance between the omega loop residues and the C3 helix was less than 1 nm. Open refers to a state in which the distance is 1.5 nm or greater.

Supplementary Table 5. Yeast strains used in this study

Name	Genotype	Reference
**PD49**	*MAT*α his3Δ1leu2Δ0 ura3Δ0 *ups1*Δ::HYG	3
**PY51**	*MAT*a his3Δ1leu2Δ0 ura3Δ0 *ups1*Δ::HYG *phb1*Δ::NAT pCM189-*PHB1*[*URA3*]	This study
**CG409**	*MAT*a his3Δ1leu2Δ0 ura3Δ0 *phb1*Δ::NAT pCM189-*PHB1*[*URA3*]	3
**CG410**	*MAT*a his3Δ1leu2Δ0 ura3Δ0 *ups2*Δ::KAN *phb1*Δ::NAT pCM189-*PHB1*[*URA3*]	3

Supplementary Table 6. X-ray crystallography data collection and structure refinement statistics

	PRELID1 wt	PRELID3b	PRELID1 K58V
**Resolution range (A)**	93.64 - 1.98 (2.051 - 1.98)	76.23 - 2.91 (3.014 - 2.91)	59.4 - 2.98 (3.087 - 2.98)
**Space group**	P 63 2 2	I 2 2 2	P 63 2 2
**Unit cell**<br>a, b, c, α, β, γ	127.31, 127.31, 177.39<br>90, 90, 120	93.02, 133.00, 146.05<br>90, 90, 90	126.00, 126.00, 178.32<br>90, 90, 120
**Unique reflections**	59554 (5844)	20180 (1989)	17719 (1728)
**Completeness (%)**	99	99.5	99.9
**I/sigma(I)**	1.73	1.56	1.37
**Wilson B-factor**	31.25	82.31	99.52
**Reflections used in refinement**	59199 (5745)	20166 (1985)	17677 (1726)
**Reflections used for R-free**	2941 (295)	969 (103)	872 (79)
**R-work**	0.2057 (0.3405)	0.2240 (0.3683)	0.2438 (0.3964)
**R-free**	0.2447 (0.3636)	0.2979 (0.4301)	0.2980 (0.4718)
**Number of non-hydrogen atoms**	4321	4519	3801
**Macromolecules**	3964	4432	3776
**Ligands**	48	48	12
**Protein residues**	493	601	496
**RMS(bonds)**	0.016	0.016	0.011
**RMS(angles)**	1.30	1.49	1.48
**Ramachandran favoured (%)**	98	95	92
**Ramachandran allowed (%)**	1.4	5.7	6.7
**Ramachandran outliers (%)**	0.21	0.3	1.2

Statistics for the highest-resolution shell are shown in parentheses.

Supplementary Table 7. Primers used in this study

Primer name	Sequence
**TL4606**	CCCGGGATGGTCCTTTTACACAAA
**TL4607**	AAGCTTTTACTCGAGGTCTTCTTCGGAAATCAACTTCTGTTCAAA CTGAGGATTTCTCGC
**TL11721**	GGCCTTCCTTCCAGTTACTT
**TL11722**	TTATGCTTCCGGCTCGTATG
**TL12090**	TCTGCACAATATTTCAAGCTATACCAAGCATACAATCAAG
**TL12091**	ATAACAATTTCACACAGGAAACAGCTATGACCATGATTAC
**TL11898 Ups1K58M F**	ACGAGGCTGTTGAAAATGTCCGGAAAGCTGC
**TL11899 Ups1K58M R**	GCAGCTTTCCGGACATTTTCAACAGCCTCGT
**TL11849 Ups1T76A F**	CCTTTTTAAGAGGTATAACAGAAGCATGGATAATCGAAGTTTCCG
**TL11850 Ups1T76A R**	CGGAAACTTCGATTATCCATGCTTCTGTTATACCTCTTAAAAAGG
**TL11900 Ups1T95S F**	CTCCACAATGAAAACTTACTCTAGGAATCTGGATCACA
**TL11901 Ups1T95S R**	TGTGATCCAGATTCCTAGAGTAAGTTTTCATTGTGGAG
**TL11904 Ups1 E108D**	GAATCATGAAGGTTGAAGATTATACTACCTATCAATTTGACA
**TL11905 Ups1 E108D R**	TGTCAAATTGATAGGTAGTATAATCTTCAACCTTCATGATTC
**TL11853 Ups1F133S F**	AGTTTTCAAGTGGCTCCAATATGGGTATCAAATCT
**TL11854 Ups1F133S R**	AGATTTGATACCCATATTGGAGCCACTTGAAAACT
**TL11906 Ups1 M135K F**	TTCAAGTGGCTTCAATAAGGGTATCAAATCTAAGGT
**TL11907 Ups1 M135K R**	ACCTTAGATTTGATACCCTTATTGAAGCCACTTGAA
**TL11896 Ups1K58V F**	ACGAGGCTGTTGAAAGTGTCCGGAAAGCTGC
**TL11897 Ups1K58V R**	GCAGCTTTCCGGACACTTTCAACAGCCTCGT
**TL12300 Ups2 I66N F**	AAGGTGTTCCCAAATGGAACATGATGATGCTAGGAGG
**TL12301 Ups2 I66N R**	CCTCCTAGCATCATCATGTTCCATTTGGGAACACCTT

**TL12302 Ups2 M93T F**	CTTAAATAAAAAGAGCTTGACCACGAGAAGCTGTAATTTAACGA
**TL12303 Ups2 M93T R**	TCGTTAAATTACAGCTTCTCGTGGTCAAGCTCTTTTTATTTAAG
**TL12310 Ups2 C101R**	GAAGCTGTAATTTAACGATGCGCAATCTATTAAAAGTCTACG
**TL12311 Ups2 C101R R**	CGTAGACTTTTAATAGATTGCGCATCGTTAAATTACAGCTTC
**TL12306 Ups2 I137T F**	TACAGCATATGGATCCACTAGAAAGCTGTGTAATAAGA
**TL12307 Ups2 I137T R**	TCTTATTACACAGCTTTCTAGTGGATCCATATGCTGTA
**TL11857 Ups2S76G F**	AGGAGGGACCAATATGGGTCATGTCAGAGAAGTT
**TL11858 Ups2S76G R**	AACTTCTCTGACATGACCCATATTGGTCCCTCCT
**TL12490 Ups2 N21F F**	CCAGGTAACCGCCGCTTTCTGGAAAAAATACCCAAATGAAATAT C
**TL12491 Ups2 N21F R**	GATATTTCATTTGGGTATTTTTTCCAGAAAGCGGCGGTTACCTGG
**TL12492 Ups2 R151W F**	TGGAAGATTGGTCAGTTCAATGGTTTTGCGAGAACGC
**TL12493 Ups2 R151W R**	GCGTTCTCGCAAAACCATTGAACTGACCAATCTTCCA
**TL12486 Ups2(60-71Ups1) F**	AGTTACTGAACGGTTGATTACTGTAAAAGGAAAGCTGCCCACAT GGGTCAAACCCTTTTTAAGAGGGACCAATATGAGTCATGTCAG
**TL12487 Ups2(60-71Ups1) R**	CTGACATGACTCATATTGGTCCCTCTTAAAAAGGGTTTGACCCAT GTGGGCAGCTTTCCTTTTACAGTAATCAACCGTTCAGTAACT
**PRELID1 K58T_F**	TCTGCTGACCactACCAATCGTATGCC
**PRELID1 K58T_R**	CGACGGCTCAGCAGTTTC
**PRELID1 K58V_F**	TCTGCTGACCGTTACCAATCGTATGCC
**PRELID1 K58V_R**	CGACGGCTCAGCAGTTTC

Supplementary References

1. Watanabe, Y., Tamura, Y., Kawano, S. & Endo, T. Structural and mechanistic insights into phospholipid transfer by Ups1-Mdm35 in mitochondria. Nat Commun 6, 7922 (2015).
2. Miliara, X. et al. Structural insight into the TRIAP1/PRELI-like domain family of mitochondrial phospholipid transfer complexes. EMBO Rep 16, 824-835 (2015).
3. Osman, C. et al. The genetic interactome of prohibitins: coordinated control of cardiolipin and phosphatidylethanolamine by conserved regulators in mitochondria. J Cell Biol 184, 583-596 (2009).
