# Supplementary material for: Structural determinants of lipid specificity within Ups/PRELI lipid transfer proteins
Source: Nat Commun. 2019 Mar 8;10:1130. doi: 10.1038/s41467-019-09089-x (PMC6408443; doi:10.1038/s41467-019-09089-x)
Supplement: Supplementary file 3 — Description of Additional Supplementary Files [file 41467_2019_9089_MOESM3_ESM.pdf]

Description of Additional Supplementary Files

File Name: Supplementary Data 1
Description: Mutations identified in reverse genetic screens, related to Figs. 2 and 5. Colored mutations are found at the residues that are mutated frequently (>5). Mutations found at Ω loop are highlighted in blue. Mutations lead to truncation are highlighted in yellow. FS; flame shift mutation, Δ; insertion of stop codon.

File Name: Supplementary Movie 1
Description: Molecular simulation of PS lipid release by the PRELID3bTRIAP1 complex. PRELID3b (cyan; Ω-loop dark blue) and TRIAP1 (green) shown as a backbone trace with anchoring residues shown as purple spheres. Bound PS shown with yellow tails, magenta phosphate group and orange serine group. Membrane lipid phosphate groups shown as light grey spheres. Second lipid occupying Site II following PS release shown in magenta.

File Name: Supplementary Movie 2
Description: Molecular simulation of PA lipid release by the PRELID1- TRIAP1 complex. Coloured as in Supplemental Movie S2. PRELID1 shown in yellow. POPA shown in dark grey with phosphate group coloured magenta.
