# Supplementary material for: Structural determinants of lipid specificity within Ups/PRELI lipid transfer proteins
Source: Nat Commun. 2019 Mar 8;10:1130. doi: 10.1038/s41467-019-09089-x (PMC6408443; doi:10.1038/s41467-019-09089-x)
Supplement: Supplementary file 7 — Reporting Summary [file 41467_2019_9089_MOESM7_ESM.pdf]

nature research

Corresponding author(s): Thomas Langer, Stephen Matthews

Last updated by author(s): Feb 5, 2019

# Reporting Summary

Nature Research wishes to improve the reproducibility of the work that we publish. This form provides structure for consistency and transparency in reporting. For further information on Nature Research policies, see Authors & Referees and the Editorial Policy Checklist.

## Statistics

For all statistical analyses, confirm that the following items are present in the figure legend, table legend, main text, or Methods section.

| n/a | Confirmed | |
|---|---|---|
| ☐ | ☒ | The exact sample size (*n*) for each experimental group/condition, given as a discrete number and unit of measurement |
| ☐ | ☒ | A statement on whether measurements were taken from distinct samples or whether the same sample was measured repeatedly |
| ☐ | ☒ | The statistical test(s) used AND whether they are one- or two-sided<br>*Only common tests should be described solely by name; describe more complex techniques in the Methods section.* |
| ☒ | ☐ | A description of all covariates tested |
| ☒ | ☐ | A description of any assumptions or corrections, such as tests of normality and adjustment for multiple comparisons |
| ☐ | ☒ | A full description of the statistical parameters including central tendency (e.g. means) or other basic estimates (e.g. regression coefficient) AND variation (e.g. standard deviation) or associated estimates of uncertainty (e.g. confidence intervals) |
| ☒ | ☐ | For null hypothesis testing, the test statistic (e.g. *F*, *t*, *r*) with confidence intervals, effect sizes, degrees of freedom and *P* value noted<br>*Give P values as exact values whenever suitable.* |
| ☒ | ☐ | For Bayesian analysis, information on the choice of priors and Markov chain Monte Carlo settings |
| ☒ | ☐ | For hierarchical and complex designs, identification of the appropriate level for tests and full reporting of outcomes |
| ☒ | ☐ | Estimates of effect sizes (e.g. Cohen's *d*, Pearson's *r*), indicating how they were calculated |

*Our web collection on statistics for biologists contains articles on many of the points above.*

## Software and code

Policy information about availability of computer code

Data collection

Gel images are acquired using Image Studio Version 5.2 (LI-COR Biosciences).
Data from plate reader was collected using Softmax Pro Version 6.3.1 (Molecular devices).
Lipidomics data were acquired by Analyst Software Version 1.6 and LipidView Software Version 1.2 (SCIEX).
Simulations were generated using gromacs version 5.
Modeller version 9. 9 was used to generate monomer models.
Coarse grained simulations used the Martini v2.2 force field and the martinize.py script .
Crystallography data were collected on the Diamond i03 and i04 beamlines using a Pilatus 6M-F.

Data analysis

Data were analyzed using Excel 2016 (Microsoft) andGraphpad Prism Version 7.03 (Graphpad Software)
Sequence alignment was generated by T-Coffee (https://www.ebi.ac.uk/Tools/msa/tcoffee/, Notredame et. al, 2000)
Simulations were analyzed using gromacs 5 code and visualised using VMD.
Crystallography data processing and analyses used the following software: Phaser MR; COOT; Phenix; Refmac5; MolProbity; xia2.

For manuscripts utilizing custom algorithms or software that are central to the research but not yet described in published literature, software must be made available to editors/reviewers. We strongly encourage code deposition in a community repository (e.g. GitHub). See the Nature Research guidelines for submitting code & software for further information.

## Data

Policy information about availability of data

All manuscripts must include a data availability statement. This statement should provide the following information, where applicable:

- Accession codes, unique identifiers, or web links for publicly available datasets
- A list of figures that have associated raw data
- A description of any restrictions on data availability

The structure data for TRIAP1-PRELID3b, TRIAP1-PRELID1 and TRIAP1-PRELID1 (K58V) complexes have been deposited in the Protein Data Bank (with PDB ID codes:

6I4Y, 6I3V, 6I3Y). Other data are available from the corresponding authors upon reasonable request. Software is obtainable via reasonable request to the corresponding author and with permission from Heidi Koldsoe (Schrodinger Inc). The source data underlying Figs 2B and 5B are provided as Supplementary Table 1 and 3, respectively. The source data of Figs. 3C, 4B, 6D, 6F and Supplementary Figures 2C, 2D, 3D, 3E, 4A, 4B, 5C, 6A-E, 6H are provided as a Source Data file.

# Field-specific reporting

Please select the one below that is the best fit for your research. If you are not sure, read the appropriate sections before making your selection.

☒ Life sciences ☐ Behavioural & social sciences ☐ Ecological, evolutionary & environmental sciences

For a reference copy of the document with all sections, see nature.com/documents/nr-reporting-summary-flat.pdf

# Life sciences study design

All studies must disclose on these points even when the disclosure is negative.

| | |
|---|---|
| Sample size | Sample size in experiments done in yeast cultures were counted as a number of experimental attempts from independent sampling of biological material. Sample size in experiments in vitro were counted as a number of experimental attempts. The statistical significance in related figures was assessed using two-tailed Student's t-test. A p values and n in column plots from Student's t test were specified in corresponding figure legend. Molecular simulations in silico: 3 replicates of each simulation composition were performed. No attempt was made to perform statistical analysis or quantify data therefore on these, the repeats were in order to confirm qualitative behaviour was reproduced. |
| Data exclusions | No data were excluded. |
| Replication | All experiments were performed initially as a pilot experiment with single or small samples and then the indications were verified experimentally by experiments with adequate sample size/replications. All attempts at replication were successful and no non-reproducible findings were observed or reported |
| Randomization | not applicable |
| Blinding | not applicable |

# Reporting for specific materials, systems and methods

We require information from authors about some types of materials, experimental systems and methods used in many studies. Here, indicate whether each material, system or method listed is relevant to your study. If you are not sure if a list item applies to your research, read the appropriate section before selecting a response.

## Materials & experimental systems

| n/a | Involved in the study | |
|---|---|---|
| ☐ | ☒ | Antibodies |
| ☒ | ☐ | Eukaryotic cell lines |
| ☒ | ☐ | Palaeontology |
| ☒ | ☐ | Animals and other organisms |
| ☒ | ☐ | Human research participants |
| ☒ | ☐ | Clinical data |

## Methods

| n/a | Involved in the study | |
|---|---|---|
| ☒ | ☐ | ChIP-seq |
| ☒ | ☐ | Flow cytometry |
| ☒ | ☐ | MRI-based neuroimaging |

## Antibodies

| | |
|---|---|
| Antibodies used | Anti-myc, Cell signaling #2276<br>anti-Cox2 yeast |
| Validation | Anti-myc https://media.cellsignal.com/pdf/2276.pdf<br>Anti-Cox2 Herrman et al., J Biol Chem 270, 27079-27086 (1995) |
